# Multi-shank 1024 channels active SiNAPS probe for large multi-regional topographical electrophysiological mapping of neural dynamics

**DOI:** 10.21203/rs.3.rs-4800131/v1

**Published:** 2024-08-07

**Authors:** Gian Nicola Angotzi, Mihály Vöröslakos, Nikolas Perentos, Joao Filipe Ribeiro, Matteo Vincenzi, Fabio Boi, Aziliz Lecomte, Gabor Orban, Andreas Genewsky, Gerrit Schwesig, Deren Aykan, György Buzsáki, Anton Sirota, Luca Berdondini

**Affiliations:** 1 Fondazione Istituto Italiano di Tecnologia, Microtechnology for Neuroelectronics Unit (NetS^3^ lab), Genova, Italy.; 2 Corticale Srl, Genova, Italy.; 3 Ludwig-Maximilians-Universitat, Faculty of Biology, Munchen, Germany.; 4 Neuroscience Institute, Grossman School of Medicine, New York University, New York, NY, USA.; 5 Department of Neurology, Grossman School of Medicine, New York University, New York, NY, USA.; 6 University of Nicosia School of Veterinary Medicine, 2414, Nicosia, Cyprus

## Abstract

Implantable active dense CMOS neural probes unlock the possibility of spatiotemporally resolving the activity of hundreds of single neurons in multiple brain circuits to investigate brain dynamics. Mapping neural dynamics in brain circuits with anatomical structures spanning several millimeters, however, remains challenging. Here, we demonstrate the first CMOS neural probe for mapping intracortical neural dynamics (both LFPs and spikes) in awake, behaving mice from an area >4 mm^2^. By taking advantage of the modularity of our SiNAPS technology, we realized an eight shanks probe with 1024 electrode channels arranged on each shank in regular arrays with an electrode pitch <30 μm. Low-noise recordings from all electrodes at 20 kHz/channel demonstrate a field of view spanning the 2D lattice of the entire mice hippocampal circuit, together with cortical and thalamic regions. This arrangement allows combining large population unit recording across distributed networks with precise intra- and interlaminar/nuclear mapping of the oscillatory dynamics.

## Introduction

Combining the two facets of single neuronal population activities and oscillatory dynamics in multichannel extracellular recordings requires high-resolution, large-scale sensing devices capable of monitoring spiking and local field potentials (LFP) within and across widely distributed circuits ^1–3^. These competing needs can be achieved by radical scaling of the number of recorded electrodes to yield dense sampling across distributed circuits. Recent studies^4–7^ demonstrated micro-/nano fabricated implantable Complementary Metal-Oxide Semiconductor (CMOS) probes with hundreds to thousands of recording electrode-pixels within small cross-sectional areas. By integrating into the same silicon substrate electrodes and electronic circuits for signal amplification, filtering and read-out, such implantable CMOS probes can simultaneously record from multiple brain regions^8,9^ with an unprecedented spatial resolution. Furthermore, the high 2D spatial resolution of these probes also permits the use of advanced automated sorting algorithms to better isolate putative single-unit activity, by exploiting correlations of individual neuron spikes measured by closely and regularly spaced electrode contacts^10–12^. Yet, current CMOS-based probe implementations are limited to linear designs whereby electrodes are arranged along the vertical/axial dimension of implantation or to designs with small longitudinal spread, thus being unable to sample anatomical structures that span several millimeters along the horizontal axis. Indeed, mesoscale sampling of the LFP using 2D CMOS-based probes with precise geometrical layout enabled insights into both intra- and translaminar features of the theta and gamma oscillatory dynamics in the hippocampus^13–16^ and neocortex^17,^ decoding of the rat position from the spatio-temporal features of theta oscillation waveshape^18^, dissecting the role of different hippocampal fields to sharp wave/ripple generation^19^ and anatomical decomposition of the extrahippocampal inputs dynamics during sleep^20,21^. Combining local and global dense recording would enable linking distributed large-scale neural ensemble sampling to the multiregional oscillatory dynamics reflected by the detailed 2D sampling of the LFP.

The ability to densely sample extracellular signals for both single neuronal populations and local field potentials for assessing inter-regional communication with appropriate anatomical resolution requires precise geometrical scaling of the CMOS-based shanks. However, positioning and inserting multiple single-shank electrode devices into the brain at a small pitch of a few hundreds of microns as required for local and global 2D sampling is a technically challenging task. Passive multi-shank silicon probes with 96 or 256 recording electrodes can sample large tissue volumes with relatively low single unit yield due to low and 1D local electrode density ^13,22^. Alternatively, active 4-shank probes (Neuropixels 2.0 ^23^ ) can achieve local dense coverage, but these probes can only record from a subset of the available electrodes simultaneously, thus limiting the monitored area and providing uneven 2D sampling at the circuit scale.

To address this general problem related to any type of brain circuits in which vertical and horizontal information needs to be simultaneously sampled, here we present a novel CMOS-probe that exploits the circuit modularity of our SiNAPS technology^7^. This allows to replicate instances of 32 electrode-pixel modules and achieve simultaneous recordings of 1024 electrode-pixels distributed on eight shanks (pitch of 300 μm), covering an area of 4.12 mm^2^ providing both dense local and large scale 2D sampling of the wide-band extracellular signal. In vivo recordings from the brain of behaving head-fixed mice demonstrate how broadband neural signals can be acquired from the entire width of the hippocampus with a vertical coverage spanning from cortical to sub-hippocampal regions. This novel method allows the monitoring of the 2D electro-anatomy of the entire hippocampus with precise region and layer resolution. At the same time, the high-density of the recording electrodes offers isolation of single neuron spikes and characterization of their spatio-temporal profiles. We demonstrate the capabilities of the multi-shank 1024 channels active probe to sample multiple layers in the hippocampus and neocortex to link electro-anatomically resolved oscillatory dynamics across the hippocampal circuits to distributed population activity of large populations of neurons.

## Results

### SiNAPS multi-shank electrode-pixel probe for large-area intracortical recordings

**Figure 1.**
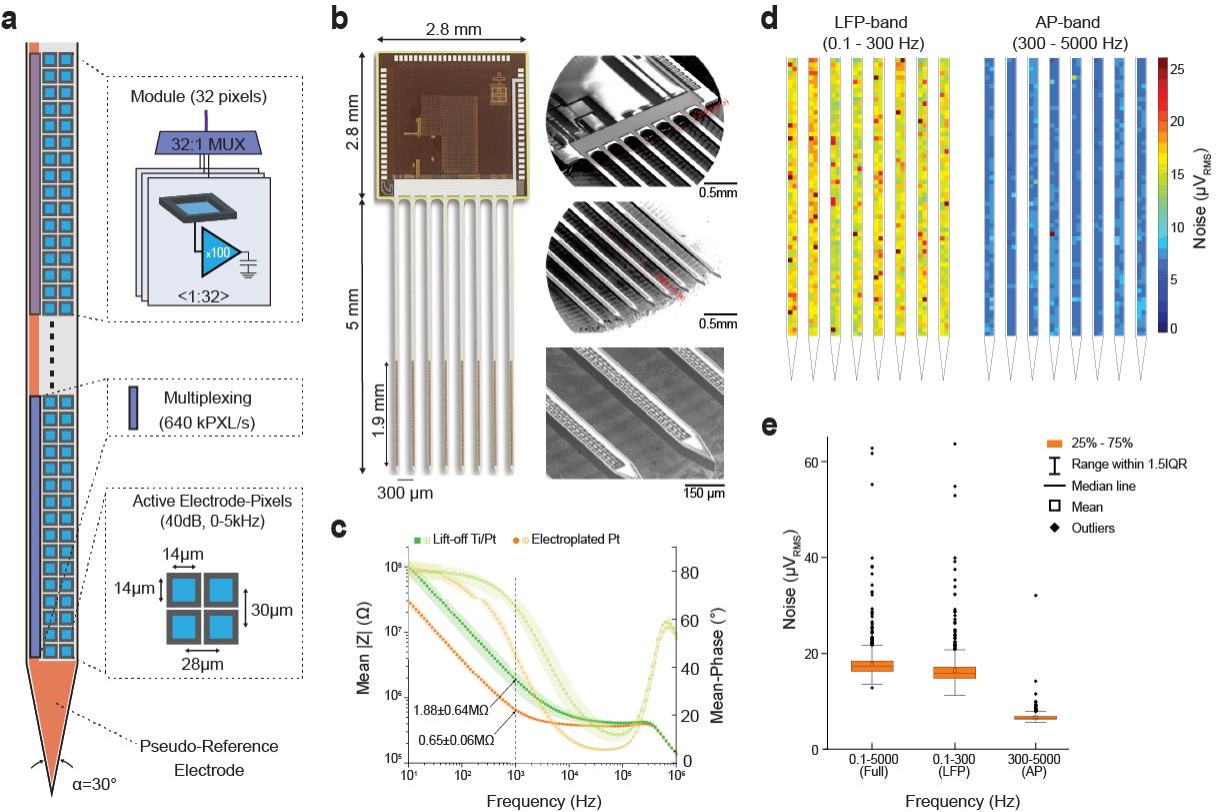
Layout and recording performances of the realized 8-shank SiNAPS probe. **(a)** Schematic view of the architecture and dimensions of a shank comprising four frontend modules of 32 active electrode-pixels arranged over a 88 μm wide shank. Each module provides a 32:1 time division multiplexer operating at 640000 electrode-pixels per second (or 640kPX/s). **(b)** Optical view and Scanning Electron Micrographs (SEM) showing a structured 8-shank SiNAPS probe at different magnification levels. **(c)** Electrochemical Impedance Spectroscopy (EIS) of platinum electrodes in saline after the microstructuring post-processing of the probes and after the electrochemical deposition of a Pt layer. The mean electrode impedance module at 1 kHz is of 1.88 ± 0.64 MΩ/electrode and is reduced to a mean impedance of 0.65 ± 0.06 MΩ/electrode after electrodeposition (n = 10 probes * 1024 electrodes, mean ± SD). **(d)** Noise of the electrode-pixel circuits measured in saline for the full recording frequency band (Full-band, 0.1–5000 Hz), the Local Field Potential frequency band (LFP-band, 0.1–300 Hz) and the Action Potential frequency band (AP, 300–5000 Hz). The false color map on the top shows the homogeneity of the electrode-pixels noise performances for the 1024 electrodes of the 8-shank SiNAPS probe. **(e)** Box-plots quantify the root-mean-square noise values of 17.78 ± 3.4 μV_RMS_ (Full-band), 16.45 ± 3.47 μV_RMS_ (LFP-band) and 6.67 ± 1.02 μV_RMS_ (AP-band) (n = 4 probes × 1024 electrodes, mean ± SD).

We realized an active dense electrode-pixel probe with 8 shanks providing continuous intracortical electrical recordings from 1024 electrodes distributed over an area of 4.12 mm^2^ (Fig 1). The CMOS circuit of this multi-shank SiNAPS probe is based on a power-efficient analog frontend electrode-pixel module that was optimized from the one proposed in Angotzi et al. (2019)^7^ and used for a single-shank probe. Each module comprises 32 electrode-pixel sites with platinum (Pt) electrode square areas of 14 μm x 14 μm and microelectronic circuits beneath each electrode for in situ amplification and low-pass filtering (Fig. 1a, Gain = 40 dB, f_−3dB_ = 5 kHz). These 32 electrode-pixels are time-multiplexed in each module to an analog single output channel. An active feedback circuit corrects for potential DC drifts that arise at the tissue-electrode interface and keeps the amplifier of each electrode-pixel within its linear range of operation. The active feedback circuit is shared in a time-division multiplexed fashion among the 32 electrode pixels of the module, and it periodically and automatically adjusts the working point of the in-pixel low-noise amplifiers. Such DC offset correction procedure was deemed favorable to integrate low-area frontends in each electrode-pixel, as it avoids the need for additional circuit elements, such as resistor-capacitor (RC) circuits or large feedback capacitors that are typically required for AC-coupled solutions. Feedback capacitors are subject to mismatch, can degrade the input impedance, and may lead to signal attenuation. Furthermore, recordings of ultraslow and DC signals may provide novel information about the physiological and pathological operations of brain circuits.

To minimize the probe width and consequent possible tissue damage, the analog frontend module arranges the 32 electrode pixels in a regular array with a center-to-center electrode pitch of ≤30 μm, and with a layout of two columns. This two-column configuration yields a cross-sectional shank width of 88 μm (Fig. 1b) and a shank thickness of 50 μm. Each shank has a length of 5070 ± 10 μm and integrates four electrode-pixel modules from the tip, for a total number of 128 electrode-pixels and an active recording length of 1924 μm. The center-to-center separation among shanks is 300 μm, tailored for covering large portions of the mouse hippocampus with an 8-shank probe defining an active recording area of 4.12 mm^2^. The electrode channel density computed by considering the overall area of this probe is close to 90 electrode-channels/mm^2^, which is an order of magnitude denser than previously published CMOS probes. The electrode impedance was reduced to 650 ± 0.6 kΩ by electrodepositing a thin rough layer of platinum (Fig. 1c). Together with circuit optimization with respect to noise, area and power consumption, we achieved a uniform low noise (Fig. 1d,e) in the action potential (AP, 0.3 – 5 kHz) and local field potential (LFP, 0.1 – 300 Hz) frequency bands accounting for 6.67 ± 1.02 μV_RMS_ and 16.45 ± 3.47 μV_RMS_ root-mean-square (RMS), respectively, while consuming only 6 μW of power per electrode-pixel. Each shank of the probe also integrates a Pt electrode covering the entire length of each shank and can be used as an on- probe pseudo-reference (or internal reference) expanding a total area for all shanks of ~ 0.4 mm^2^.

### In vivo validation of the recording system and estimation of the signal quality

**Figure 2.**
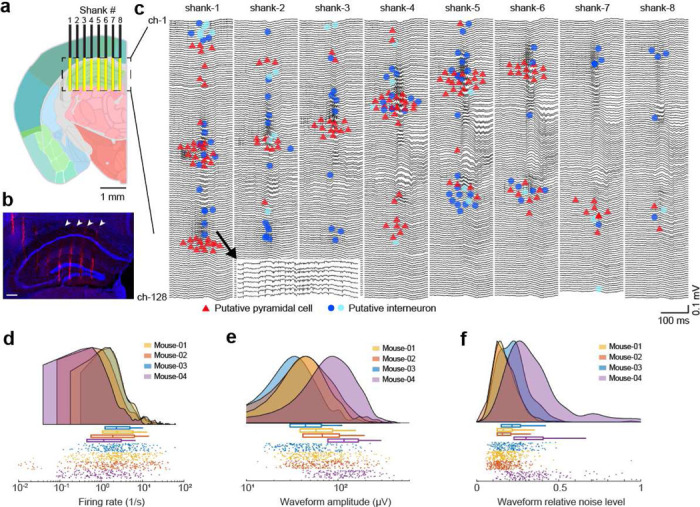
Multi-regional recording in head-fixed mice using SiNAPS electrode. **(a)** The schematic of the ideal recording location in the hippocampus is overlaid on image 73 of the Allen Mouse Brain Atlas. The yellow area shows the location of recording electrodes spanning 2141 by 1924 μm (width, depth). **(b)** Histological reconstruction of the probe tracks with 4′,6-diamidino-2-phenylindole (DAPI; blue) and DiI (red) staining. White arrows show shanks 4–8. Scale bar is 250 μm. **(c)** Wide-band signal (0.1 – 5000 Hz) recorded on 1024 channels in a wild-type mouse. The putative location of recorded neuron somata is overlaid on the LFP signal (n = 147 putative pyramidal cells, 94 narrow interneurons and 19 wide interneurons represented by red, blue, and cyan, respectively). Neurons were clustered in the cellular layers of the cortex and hippocampus (ch-1 represents the dorsal surface of the brain). Zoomed inset shows the spiking activity from shank-1. **(d-f)** Firing rate (**d**), waveform amplitude (**e**; trough-to-peak), and relative noise level (**f**; waveform standard deviation divided by the waveform amplitude) of hippocampal single units (n = 4 mice, one session per mouse).

**Figure 3.**
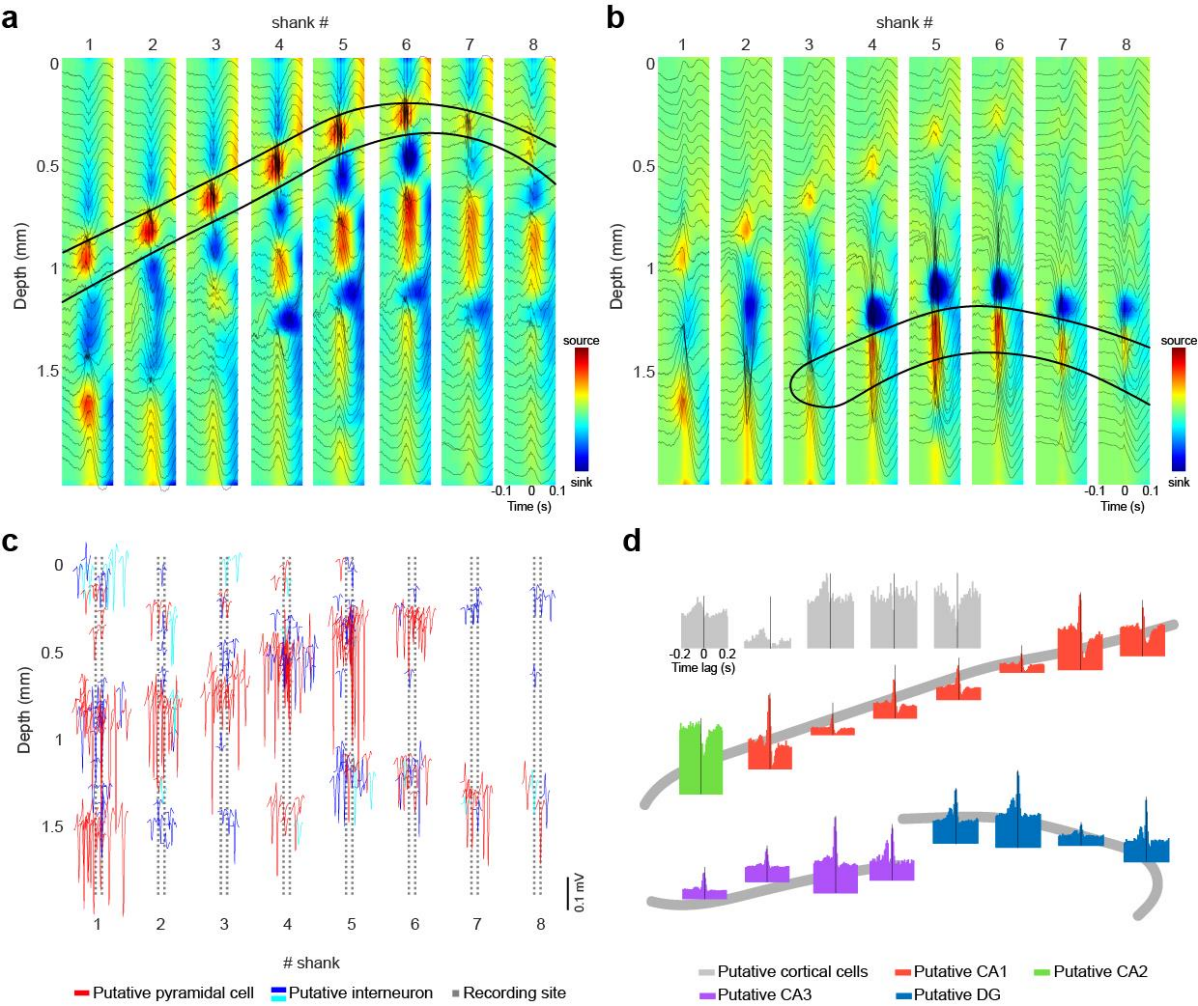
Electroanatomy of the hippocampus. **(a)** Sharp wave ripple triggered LFP-CSD map (n = 100 ripples, n = 64 channels per column of a shank). The average LFP signal of 32 channels (every second channel in one column) is overlaid on the CSD map. Note the characteristic negative SPW and sinks in the stratum radiatum accompanied by ripples and flanked by passive sources in the pyramidal layer and stratum lacunosum-moleculare after the ripple peak. **(b)** Dentate spike triggered LFP-CSD map (n = 542 dentate spikes, n = 64 channels per column of a shank). The average LFP signal of 32 channels is overlaid on the CSD map. Note the characteristic source-sink-source configuration. Zero millimeter corresponds to the top most channel on each shank. **(c)** Simultaneously recorded well isolated single units recorded with SiNAPS electrode from the hippocampus of a head fixed, awake mouse. The mean waveform of each neuron is shown at the location of the maximum waveform amplitude (n = 260 putative single units, red is putative pyramidal cell, blue is putative narrow waveform and cyan is putative wide waveform interneuron). Zero corresponds to the location of the topmost channel of the shank. **(d)** Separation of CA2 region based on peri-ripple firing rate histograms of single units (ripples were detected in the pyramidal layer of CA1). Histograms are color coded to mark their regions of origin. Each histogram corresponds to a cluster of single units in **c**. Hippocampal histograms are remarkably similar with the exception of CA2 cells, allowing identification of this subregion.

To demonstrate the capabilities of the SiNAPS technology, we performed recordings in head-fixed awake mice (n = 7 mice, **Suppl. Fig. 1 and 2**). The SiNAPS probe was inserted either in the hippocampus targeting CA1, CA3 and dentate gyrus subregions and the overlying cortex simultaneously (Fig. 2 a,b) or in the frontal cortical areas targeting prelimbic, cingulate and motor cortices (**Suppl. Fig. 3**). Laminar LFP recordings allowed the identification of cell body and dendritic layers based on established electrophysiological markers (Fig. 2c, Fig. 3a,b). In addition to wide-band LFPs, the low impedance electrodes provided high signal-to-noise recordings of extracellular spikes (82 ± 64 μV peak-to-peak voltage, mean ± SD; n = 5 mice, Fig. 2d-f). The high-density of electrode channels enabled the recording and separation of spikes from individual neurons on multiple electrodes simultaneously (4–6 electrodes typically). This redundancy in the recorded signals can be exploited to improve the quality of spike sorting. After automatic clustering of recorded spikes using KiloSort2.5^24^, we isolated 1300 neurons across 5 recording sessions (260 ± 94 single units/session, mean ± SD; n = 5 mice). These units were classified into putative cell types based on waveform and spike train characteristics (Fig. 2c, see Methods for criteria of single unit classification).

To obtain valid LFP measurements, most neuroscience experiments use a reference screw above the cerebellum, considered to be the most electrically neutral part of the brain. We tested both platinum wire and stainless steel screw as reference electrodes and found that the signal quality did not depend on the material of the reference electrode. We also compared these external versus internal pseudo-reference in the same animal (**Suppl. Fig. 4a**). Using the internal reference, each shank of the SiNAPS probe can act as a reference, creating a reference plane with a total area of 0.4 mm^2^. The single unit yield was similar regardless of the reference configuration (n = 57 and 58 single units using external and both references, respectively). We also tested whether the internal reference configuration could distort the LFP signal using visual evoked potentials. Recordings from primary and secondary visual cortices showed similar evoked responses and current sink and source distributions regardless of the reference choice (**Suppl. Fig. 4b,c**).

### Electroanatomy of hippocampal layers

The SiNAPS probe was designed to optimally record 2-dimensional (2D) laminar snapshots from brain structures such as multiple cortical or hippocampal layers. The recording channels span a brain area as large as 4.12 mm^2^ (2141 μm × 1924 μm). Simultaneous 1024 recording channels across 8 shanks allow the decomposition of the LFP signal into its individual sources. The relationship between afferents to dendrites and somata in the hippocampus and the characteristic depth profiles of various oscillatory LFP patterns can be used to identify hippocampal layers and their transitions^25^. First, we detected sharp-wave ripples (SWR) in the pyramidal layer of CA1^26^ and constructed a ripple-triggered average current source density (CSD) map (Fig. 3a)^27^. The 2D CSD map confirmed the source-sink-source distribution of SWRs corresponding to apical dendritic excitation of CA1 pyramidal neurons by the synchronous activity of CA3 neurons. To further differentiate the subregions of the hippocampus, we identified a channel in the hilus and in the molecular layer of the dentate gyrus manually and detected LFP dentate spikes (DS) automatically^28^. We constructed a DS-triggered average current source density (CSD) map which confirmed the source-sink-source configuration of the medial perforant pathway input from the medial entorhinal cortex innervating the dendrites of granular cells (Fig. 3b). Utilizing the high yield of single units, we confirmed the soma location of the recorded neurons, estimated by the peak-to-peak waveform amplitude of each unit^13,29^ (Fig. 3c) and assigned to the recording electrode-pixel of the relevant shank in the CSD map. The high number of single units also confirmed that large fractions of both pyramidal cells and interneurons fired synchronously during SWRs in the CA1 subregion but less so in the CA2 subregion^19^ (Fig. 3d).

An alternative way to construct the electroanatomic map of the hippocampus is to exploit the observation that gamma oscillations are layer-specific in the hippocampus and neocortex^16,22.^ Within-layer gamma coherence is always larger than across different layers due to the projection of the rhythmic inputs from upstream partners. Coherence maps in the broad gamma frequency band (30 – 90 Hz) were constructed between LFPs at manually selected electrode-pixels in different layers (selected electrode-pixels were chosen from cortex, stratum oriens, pyramidale, radiatum of CA1 and stratum pyramidale of CA3, granule and molecular cell layer of DG) and the remaining 1023 channels. This procedure reliably outlined the anatomical boundaries in cortex and subregions of the hippocampus (**Suppl. Fig. 5**) and provided a 2D anatomical map of the hippocampus in vivo. The electroanatomic map constructed from combined unit firing and LFP signals corresponded faithfully to the histological reconstruction of the electrode tracks and the anatomic layers of the hippocampus (Fig. 2).

**Figure 4.**
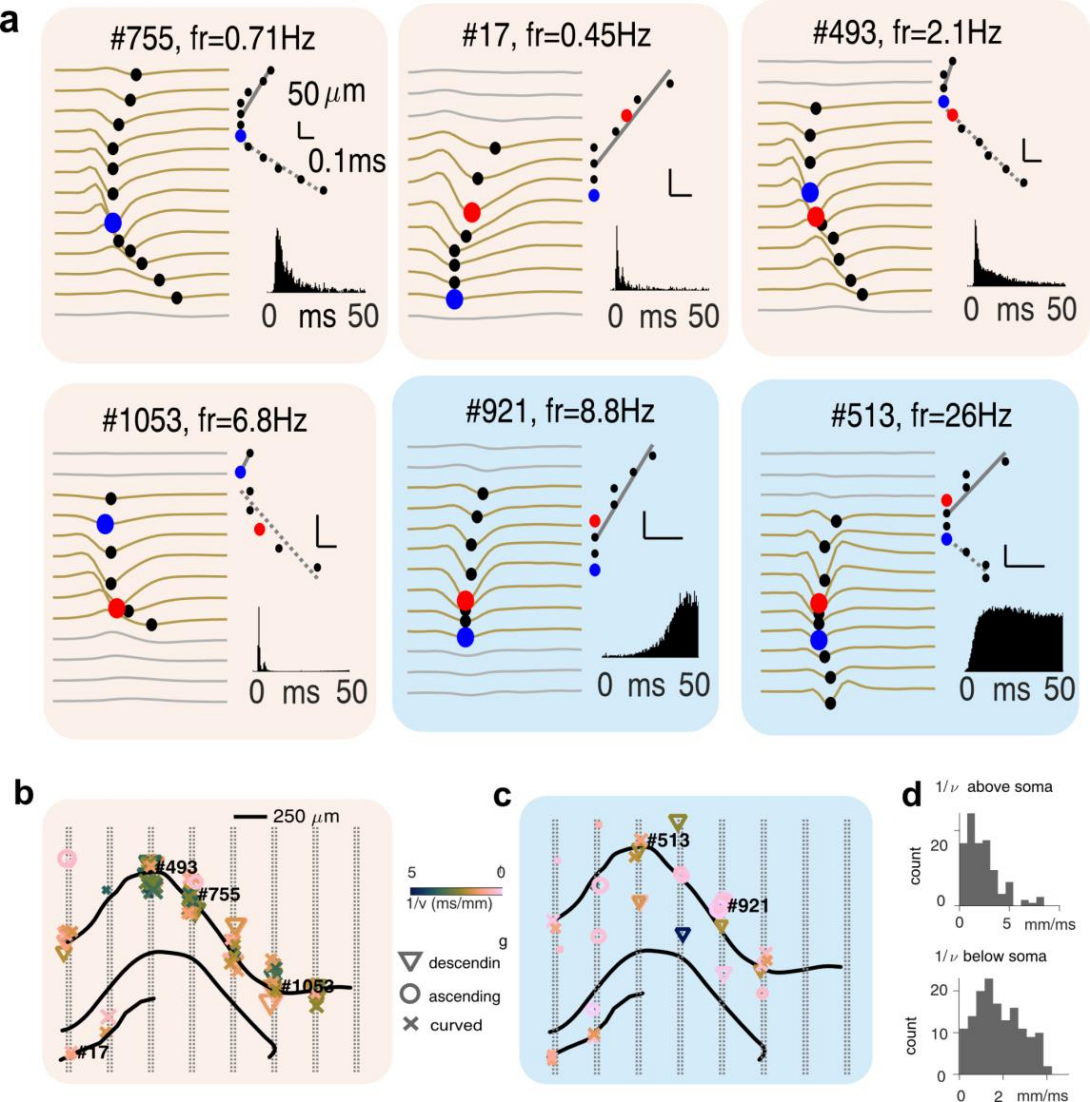
Topographic analysis of the action potential backpropagation. **(a)** Examples of backpropagation of the action potentials (AP) in 4 putative pyramidal (orange shaded panels) and 2 putative inhibitory cells (blue shaded panels). Each panel shows the average AP waveform across channels surrounding the approximate soma location (left, black markers show spike troughs, red markers show troughs of the largest amplitude and blue markers show the earliest spike trough), quantification of the AP backpropagation speed and direction (right top) and the spiking autocorrelogram (right bottom). **(b)** Depiction of the SiNAPS 8-shank probe (gray dots) and the approximate anatomical location of the hippocampal layers (black curves) from an example recording session. Superimposed are approximated soma locations of all well-isolated putative pyramidal cells (left, orange shading, 118 cells) and inhibitory cells (right, blue shading, 38 cells) with different backpropagation characteristics (marker symbol: direction of backpropagation; color: inverse speed and marker size: spatial extent of detected backpropagation). **(c)** Population summary distributions for inverse speed of AP backpropagation above (top) and below (bottom) the putative soma location.

To further exemplify the opportunities afforded by the dense and large area sampling of the neural tissue, we characterized patterns of action potential (AP) backpropagation across all hippocampal layers sampled by the 8-shank SiNAPS probe (Fig. 4a-c). Such analysis can assist in further sub-categorizations of single unit clusters beyond the pyramidal vs interneuronal putative labels by revealing their main dendritic orientation^30^. Using the channel with the largest AP amplitude as the reference channel, a search was performed for above-threshold troughs (> 12% of the actual peak) on nearby electrodes (± 250 μm)^31^. Using the relative distance of the recording channels and the trough delays relative to the earliest trough, two linear fits were extracted for each cluster, one above and one below the putative soma location. The channel with the earliest peak was defined to be the putative AP generation site (typically the axon hillock). In many cases the earliest trough did not coincide with the largest amplitude AP, the latter being the conventionally assigned soma location. This may be explained by the axon hillock-initiation area, producing an earlier depolarisation trough which is shortly followed by the full membrane depolarisation event. Using these linear fits, we classified backpropagation as ascending, descending, and or both (we termed as curve); a classification that may correlate with the underlying dendritic tree, eg., apical and basal dendrites or both. Further characteristics of the AP backpropagation, such as the behavior-dependent variation of spatial extent and the velocity of the backpropagation might offer important information about in vivo dendritic electrogenesis^32,33^(Fig. 4d).

**Figure 5.**
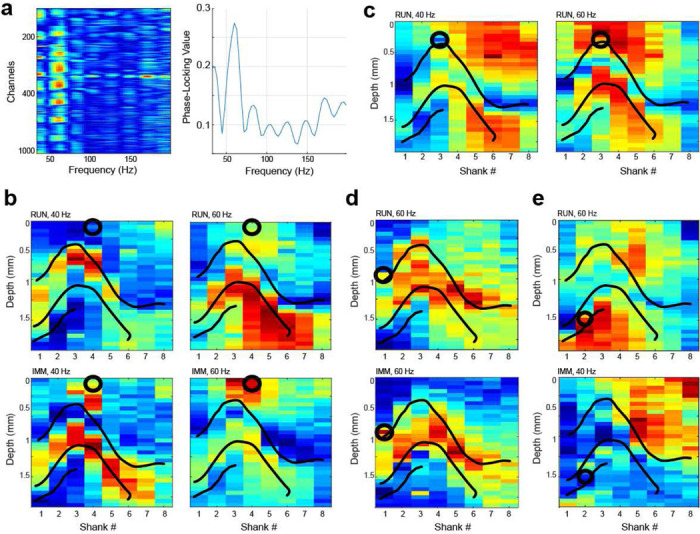
Topographic analysis of the phase-locking of unit activity to the LFP. **(a)** Example of the pseudo-color-coded strength of phase locking value (PLV) of a single interneuron (str. oriens) spiking to the LFP across all channels of the array (left) and to a best-locked channel in CA1 str. rad. (right). **(b-e)** Example color-coded topographic PLC at various peak frequencies and different brain states for example neurons. Superimposed contours represent locations of the CA1 stratum pyramidale, DG granular layer and CA3 stratum pyramidale. Black circle shows the approximate location of the unit cell body. Zero millimeter corresponds to the top most channel on each shank. **(b)** PLV map of CA1 str. oriens putative interneuron at 40 Hz (left) and 60 Hz (right) during running (top, RUN) and immobility (bottom, IMM) states. **(c)** PLV map of CA1 putative pyramidal cell at 40 Hz (left) and 60 Hz (right) during running state. **(d)** PLV map for putative CA1 pyramidal cell at 60 Hz during running (top) and immobility (bottom) states. **(e)** PLV map for putative CA3 pyramidal cell at 60 Hz during running (top) state and 40 Hz during immobility (bottom) state.

Finally, we set out to utilize the two main design advantages of the SiNAPS arrays that stem from dense large-scale 2D sampling, namely, the ability to isolate distributed anatomically-localized neural ensembles and extract electro-anatomical properties of oscillatory dynamics from 2D-sampled laminar-resolved LFP signal. To this end, we explored laminar diversity of the hippocampal gamma oscillations through the prism of phase coupling of the spiking of hippocampal populations to the LFP signal across the array^13,16,17,34,35.^ Laminar-localized oscillatory power in the gamma band of the LFP reflects synchronous synaptic inputs of the local or upstream gamma rhythm generators^16,17,34^ (**SFig. 4**), which results in phase-coupling of the firing of neural ensembles both upstream, as part of the generator circuit, or downstream, as part of post-synaptic population that is entrained by these gamma-rhythmic inputs^35^. Here we illustrate this approach to study circuit-specific gamma oscillations via both pre- and post-synaptic populations that they entrain by computing the strength of phase-locking of the firing of diversely located populations of single neurons to the LFP across the whole hippocampal proper during both locomotive and immobile states (Fig. 5). Single neurons show variable decree of phase-locking (PLV) of their spiking to the LFP signal across the array and gamma frequencies (Fig. 5a), which reflects on the anatomical origin of the gamma oscillations observed in the LFP. Anatomical maps of the PLV at distinct peak PLV frequencies reveal laminar-specific profiles that can be similar or different across frequencies and states (Fig. 5b), showing similar or different gamma generators that these neurons are recruited by at the respective frequency and state. We observed a variety of motifs with some neurons phase-locking to distinct laminar patterns that are different between states at one frequency (Fig. 5b, left) and similar at the other (Fig. 5b, right). A single neuron could be coupled to the LFP in multiple anatomical domains and magnitude of coupling could vary across these domains depending on the oscillation frequency (Fig. 5c), be similar (Fig. 5d) or combine common and different domains across frequency and state (Fig. 5e). Putative excitatory and inhibitory neurons localized in distinct hippocampal fields thus reflect through these anatomical domains of their phase-coupling the “dictionary” of circuit-specific gamma oscillations, that emerge transiently and recruit specific populations across all layers. Collectively obtaining these common anatomical motifs allows for extracting associated distributed populations that will enable uncovering the origin of the generator and the target populations it entrains^35^.

## Discussion

As demonstrated in this work, SiNAPS probes with 1024 electrode-pixels distributed across eight equally spaced shanks can record LFPs and APs concurrently from a large active recording area of ~4 mm^2^. This expansive spatiotemporal sampling of large brain areas yields a finer description of neural dynamics and population activity of single units across different brain regions and subregions while overcoming the drawbacks of passive neural probes such as large footprint, wiring and low-density local sampling that is suboptimal for spike sorting ^13,22,36^. Here, we demonstrated the unique strength of SiNAPS multi-shank probe recordings to study the close relationship between mesoscopic network dynamics, population activity of single neurons and anatomy in the hippocampus. By covering the mice hippocampus, these high-resolution electrical recordings can be used to generate extremely detailed 2D circuit-level electro-anatomical maps of the hippocampal neural dynamics and anatomically-resolved characteristics of the spatio-temporal backpropagation of individual neurons. To illustrate the advantage of joint dense local and global sampling by the SiNAPS probe we characterized the topography of the gamma oscillatory dynamics via analysis of the phase locking of pyramidal and interneuron units activity to LFPs for different frequencies and behavioral states.

A number of different approaches have been recently proposed to target single neuron recordings from brain circuits with sub-millisecond temporal resolutions. High channel count solutions based on the use of thin, flexible multi-electrode polymer probes were recently proposed^37,38^, but their efficacy remains to be demonstrated. In addition to clear advantages, those approaches are less ideal for several research goals, which require knowledge of the precise geometric relationships among all recording sites to attribute neural sources of neural dynamics to the boundaries of regions and sub-regions in layered or deep structures such as neocortex or hippocampus and to examine functional connections within-layer, across-layers, and across-regions with accurate coregistration to their anatomy^14,16,17,19,22,39^.

Active implantable probes based on CMOS technology surpass limitations of passive probes, including vulnerability to electromagnetic interference, signal attenuation due to parasitic capacitances, and crosstalk between adjacent channels arising from densely packed metal lines. These include Neuropixels^4^, Neuroseeker^5^ and the SiNAPS active probes^7^. SiNAPS probes overcome the major scaling bottleneck caused by the spatial limits of analog front ends^8^, thus allowing on-probe multiplexing over modules of 32 in-situ (i.e beneath each electrode) amplified and filtered electrode signals using a few, low impedance output lines robust to electromagnetic noise and cross-talks. This approach has the advantage of allowing the simultaneous, full-band sampling of all 1024 channels. By increasing the number of recording sites while minimizing overall probe size, particularly the base area, SiNAPS is also a promising solution for future chronic experiments in small laboratory animals, such as mice.

Similar to Neuropixels and Neuroseeker devices, SiNAPS probes also use CMOS technology to integrate dense arrays of electrodes and read-out circuits. However, SiNAPS grounds on a distinct circuit solution that uses the Active Pixel Sensor (APS) concept originally developed for image sensors^40^, whereby active circuits for signal amplification, low-pass filtering, and read-out are located directly underneath each electrode-pixel^41^. This leads to distinct advantages. Notably, the SiNAPS approach allows integrating an equal number of front ends and electrodes as required for simultaneous recordings from the entire electrode array in contrast to other technologies where only a subset of channels can be simultaneously sampled. Consequently, the continuous and multi-regional recording of both single neuronal activity sampled by the array and LFP dynamics is ensured at the level of each single trial and simplifies data analysis. In addition, the unique modularity of the circuit architecture of SiNAPs probes allows the number of electrodes, shaft configurations from single to multiple shafts, and probe geometries to be scaled by replicating modules of 32 closely spaced electrode pixels, rapidly extending probe capabilities to meet specific experimental needs.

The multi-shank SiNAPS technology advances the current tools that have been developed over the last few years for the post-processing and interpretation of the rich spatio-temporal content of data collected from high-density neural probes^10–12,23,42,43^. Ultimately, this will lead to a better understanding of the circuit-level mechanisms underlying distributed brain functions. In this work, we mainly used recordings from the hippocampus to illustrate the benefits of the new technology because of its well-known laminar structure and a large body of background knowledge on both the population activity of single neurons and the large-scale network dynamics, making it ideal to reveal the link between the two. We demonstrate the power and utility of 2-D recordings for precise layer identification and inter-regional communication. The large number of anatomically resolved and well-isolated putative pyramidal cells and interneurons offer inter-regional analyses of input-output circuit operations. One such promising method is relating spiking activity to the 2-D LFP data, potentially understanding the link between ensembles of single neurons and oscillatory dynamics within and across hippocampal regions in behaving animals. We also show examples of cortical recordings and illustrate how a similar approach can be applied to discovering principles of interlayer and inter-regional communication in the neocortex. The large span of the probe shanks also offers opportunities to study temporal interactions among distinct cortical columns in sensory areas, multiple subcortical regions and nuclei where dense 2D electroanatomy allows online demarcation of the anatomical boundaries, such as in thalamic, basal ganglia and brain stem nuclei^44–46^. Overall, SiNAPS probes can explore the close relationship between distributed multiple single-unit population activity, mesoscopic network dynamics and functional connectivity.

## METHODS

### Fabrication of SiNAPS probes

The multi-shanks SiNAPS probes were realized in a standard 180 nm Complementary Metal-Oxide Semiconductor (CMOS) technology. The microstructuring process was first established for 4-shanks probe prototypes at dye level and then scaled to wafer-level post-processing with similar processes (Corticale Srl, Italy) for 8-shanks probes. Prototyping devices were delivered from the CMOS foundry as single dies with the size of (W×L×T = 3 mm × 8.5 mm × 152.4 μm) and were post-processed in the IIT cleanroom facility to modify the electrode material and to structure thin probes. The first post-processing step is used to structure a noble metal (Pt) layer on top of the native CMOS metal of each electrode-pixels. The top CMOS insulator is opened at a size of 10 × 10 μm^2^, and the Pt layer is structured with a size of 14 × 14 μm^2^ to guarantee complete coverage. The second post-processing step uses reactive ion-etching of silicon to structure the probe shape and a backside grinding process to thin the probes to a final thickness of 50 μm. To reduce the electrode impedance, a rough layer of Pt was electroplated on the Pt electrodes using a potentiostat/galvanostat (PG-STAT204, Metrohm Autolab, Switzerland) and a three electrodes electrochemical cell (Ag/AgCl reference electrode, Pt counter electrode and the short-circuited 1024 electrodes of the SiNAPS probe as working electrode). Electrochemical stripping in sulfuric acid (0.5 mol/l) is used to clean the electrodes. Then, Pt is electroplated using a commercial solution (“Platinum AP + 4G/L Pt”, Technic, Italy) and by applying a current of 10 nA/electrode for 1 h. After post-processing SiNAPS probes were mounted on a printed circuit board (PCB) and wire bonded for interfacing with the SiNAPS recording system.

### Electrical and Electrochemical characterization

Prior to in vivo recordings, the realized SiNAPS probes were tested with respect to their electrical and electrochemical performances. For the electrical characterization, the noise contribution of the electronic circuits is measured in dry conditions by setting the switch that is integrated in each electrode-pixels to connect all the electrodes to a common ground. The noise of the electrode-pixels was then measured in wet conditions by dipping the SiNAPS probes in a beaker containing a saline solution (NaCl 0.9%). For both conditions, data collected at the output of the whole signal acquisition chain of the probe is used to compute the power spectral density from which we quantified the root mean square noise contribution within specific frequency bands.

Electrochemical impedance spectroscopy (EIS) was used to quantify the electrode impedance in saline solution using a potentiostat/galvanostat (PG-STAT204, Metrohm Autolab, Switzerland). The impedance value for the single electrode can be estimated as 1024× larger than the one measure and three electrodes electrochemical cell (Ag/AgCl reference electrode, Pt counter electrode and the short-circuited 1024 electrodes of the SiNAPS probe as working electrode). The module of the impedance for a single electrode at 1 kHz was then computed by dividing the measured value by the number of electrodes.

### SiNAPS data acquisition system

Electrophysiological data were collected from SiNAPS probes using two different instruments for experiments performed at LMU and NYU, respectively. In the first case, we used the SiNAPS Research Platform (IIT, Italy), while in the second case, a commercially available data acquisition system (SmartBox Pro, Neuronexus, USA). The use of two different systems in different laboratories strengthens the reproducibility of the SiNAPS probes’ recording performances.

#### Experiments at LMU.

These experiments were performed using the SiNAPS Research Platform (**Suppl. Fig. 6**), which includes a data acquisition board, a digital control unit, a custom coax cable assembly with embedded electronics, and data acquisition software running on a PC equipped with a Camera Link frame grabber (Grablink Base, Euresys, Belgium). The data acquisition board provides a bank of 32 analog to digital converters (MAX11105, Maxim Integrated, USA, 20 kSample/s, 12-bit resolution over a range of 2.4V), one for each of the 32 electrode-pixels modules of the SiNAPS probe. The digital control unit is built on an Opal Kelly ZEM4310 integration module based on an Altera Cyclone IV FPGA. The firmware of the digital control unit ensures the correct operation of the SiNAPS probe and data communication. This includes the SiNAPS IP core (Corticale, Italy) that provides control signals, a bank of programmable bandpass filters (second order FIR filters with programmable high pass cut-off of 2 Hz or 300 Hz and fixed low pass cutoff frequency of 5kHz) and an automated calibration procedure to set the optimal voltage biasing^47^ of the SiNAPS probes prior to recordings. The custom coax cable assembly (1m long) connects the data acquisition board with the head-stage mounting the SiNAPS probe. This assembly also integrates components for supplying the 1.8V nominal power to the probe and includes a multichannel digital to analog converter (12 bit over 1.8V range) to generate the analog voltage biases set by the calibration procedure. The PC runs data acquisition software (SiNAPS Control Software, IIT, Italy) for data visualization and storage. This was developed in Microsoft Visual Studio Enterprise 2019 (Ver. 16.11.36, Microsoft Corp, USA) using the general-purpose high-level C# programming language and the .NET Framework 4.8.1 (Microsoft Corp, USA). The SiNAPS Research Platform concurrently stores electrode-pixel data processed in real-time by the FPGA, as well as a datastream of unprocessed raw data. This raw data is used for offline signal processing to obtain electrode signals with a frequency bandwidth ranging from 0.1 Hz to 5 kHz.

#### Experiments at NYU.

Experiments were performed using the commercial system available from Neuronexus implements similar functionalities through the SmartBox Pro and the SiNAPS Interface Box (https://www.neuronexus.com/products/sinaps-interface-box/) which are interconnected through a standard HDMI cable. As for the SiNAPS Research Platform, data from each available electrode-pixel is acquired and digitized at 20 ksample/s, with a 12-bit resolution over a 2.4 dynamic range through the SiNAPS Interface Box which also implements the same SiNAPS IP core (Corticale, Italy). A single HDMI cable is used to transmit in real time the digitized and filtered data from the SiNAPS BOX Interface to the SmartBox Pro which, in turn, implements a high-rate data transmission to the PC, where data is stored, analyzed and visualized by the Allego Software (Neuronexus, USA).

### Animal experiments

#### Experiments at NYU.

All experiments were performed in accordance with the Institutional Animal Care and Use Committee at New York University Medical Center. All efforts were made to minimize the number of animals used and the incurred discomfort. Animals were handled daily and accommodated to the experimenter before the surgery and head-fixed recording. Mice (adult male n = 5 C57/Bl6 mice, 26–31 g) were kept in a vivarium on a 12-hour light/dark cycle and were housed two per cage before surgery and individually after it. Atropine (0.05 mg kg–1, s.c.) was administered after isoflurane anesthesia induction to reduce saliva production. The body temperature was monitored and kept constant at 36–37 °C with a DC temperature controller. Stages of anesthesia were maintained by confirming the lack of a nociceptive reflex. The skin of the head was shaved, and the surface of the skull was cleaned by hydrogen peroxide (2%). A custom 3D-printed headpost^48^ (Form2 printer, FormLabs, Sommerville, MA) or aluminum headpost and 3D-printed recording chamber (LMU) was attached to the skull using C&B Metabond dental cement (Parkell, Edgewood, NY). The location of the craniotomy was marked and a stainless-steel ground screw with header pin was placed above the cerebellum. Each animal recovered for at least 7 days prior to habituation of the head-fixation. Animals were allowed to walk freely either on a treadmill (NYU, **Suppl. Fig. 1a**, https://github.com/misiVoroslakos/3D_printed_designs/tree/main/Treadmill_Rinberg) during recording sessions. The day before recording, a craniotomy was performed (2 mm posterior from Bregma and 1.5 mm lateral to midline targeting hippocampus and 2 mm anterior from Bregma and 0.5 mm lateral to midline targeting midline cortices) and the dura was removed. After the surgery, the craniotomy was sealed with Kwik-Sil (World Precision Instruments, Sarasota, FL) until the recording. On the day of the recording the animal was head-fixed, the craniotomy was cleaned and the headpost was filled with sterile saline. The ground of the probe PCB was connected to the header pin and the probe was inserted to the target depth using a manual micromanipulator (MM-33, Sutter Instruments, Novato, CA). We constantly monitored the electrophysiological signal and transistor bias during insertion. The collected data was digitized at 20 kS/s using Smartbox Pro and Activus SiNAPS interface box and visualized using Radiens^™^ Allego software (NeuroNexus Technologies, Ann Arbor, MI) (NYU). We waited at least 30 minutes after reaching the target depth before starting the data acquisition. 2-hour sessions were recorded from each mouse. After the recording session, the craniotomy was sealed with Kwik-Sil, and the animal was put back into its homecage. If more than one session was recorded from an animal, a new craniotomy was prepared as described above on the contralateral side.

#### Experiments at LMU.

All experiments were performed in accordance with the European Communities Directive 2010/63/EC and the German Law for Protection of Animals (TierSchG) and were approved by local authorities (ROB-55.2–2532.Vet$_$02–16-170). Animals (C57/Bl6 mice, n = 2, 3–5 months of age) have been trained to navigate a virtual linear track to receive a 5 μl sucrose/water (1% w/v) reward after each trial completion (**Suppl. Fig. 2**). Once stable performance was achieved, the animals underwent stereotaxic surgery. Following deep anesthesia with MMF (Medetomidine, Midazolam, Fentanyl) and isoflurane (0.5% v/v in oxygen) the animals were equipped with a custom, lightweight, 3D-printed recording chamber above the exposed skull (sealed with a thin layer of dental acrylic) as well as an aluminum head-post. Stainless steel and Ag/AgCl ground/reference electrodes were placed above the cerebellum and PFC. Additionally, a 2.2 mm long and 3.5 mm wide cranial window above the left hippocampus (AP −2.3 mm) was established, the dura was removed and the window was sealed with Kwik-Cast (World Precision Instruments Germany GmbH). During the surgery, the body temperature was monitored and kept constant at 37.5 °C using a homeothermic blanket. The animals were allowed to recover for at least 7 days (3 days of post-operative care, Enrofloxacin and Metacam) with food and water ad libitum. At the day of the recording, the animals were placed on the running wheel and their head was kept firmly via the head-post and an additional adjustable articulated mount attached to the front of the recording chamber. The Kwik-Cast seal was carefully removed, and the implantation site was inspected for possible re-growth. The exposed brain surface was kept moist with warm saline. The SiNAPS probe was lowered using a linear nanopositioner (PI Q-531.330 and PI E-873 Q-Motion Servo Controller) under tight visual control at a speed of 2–5 μm/s. The probe’s position was allowed to settle for at least 20 minutes, before bias voltage adjustments could begin. The bias voltage was set to the highest possible value where no channel was saturated and subsequently decreased again by 5–10 mV. If after 15 minutes of waiting time, the signal quality required additional gain adjustments, this step was repeated, otherwise the recording was started. The collected data was digitized at 20 kS/s using custom acquisition hardware and software.

### Visual evoked potential in head-fixed mice

The pupil was dilated using 1% atropine sulfate eye drops. The room lights were turned off to allow dark adaptation for 20 minutes. 5 ms light pulses were delivered using an LED light source (#Fortoo-12V-PMMA008, Fortoo) and 200 light pulses were delivered at 0.2 Hz using an Arduino UNO. The visual stimuli were delivered while the electrophysiology signal was collected using internal reference and then external reference configuration. The probe was kept at the same dorsoventral location for both measurements.

### Single unit analysis

A concatenated signal file was prepared by merging all recordings from a single animal from a single day. To improve the efficacy of spike sorting, stimulation-induced onset and offset artifacts were removed before automatic spike sorting (1 ms before and 5 ms after the detected artifacts, linear interpolation between timestamps). Putative single units were first sorted using Kilosort 2.5^23^ and then manually curated using Phy2 (https://phy-contrib.readthedocs.io/).

### Cell type classification

In the processing pipeline, cells are classified into three putative cell types: narrow interneurons, wide interneurons, and pyramidal cells. Interneurons are selected by 2 separate criteria; narrow interneurons are assigned if the waveform trough-to-peak latency is less than 0.425 ms. Wide interneuron is assigned if the waveform trough-to-peak latency is more than 0.425 ms and the rise time of the autocorrelation histogram is more than 6 ms. The remaining cells are assigned as pyramidal cells. Autocorrelation histograms are fitted with a triple exponential equation to supplement the classical, waveform feature-based single unit classification (https://cellexplorer.org/pipeline/cell-type-classification/)^29^. Bursts were defined as groups of spikes with interspike intervals < 9 ms.

### Local field potential analysis

Ripple detection and wavelet spectrogram calculation were performed as previously described^26^. To detect ripples a single electrode in the middle of the pyramidal layer was selected. The wide-band LFP signal was band-pass filtered (difference-of-Gaussians; zero-lag, linear phase FIR), and instantaneous power was computed by clipping at 4 SD, rectified and low-pass filtered. The low-pass filter cut-off was at a frequency corresponding to p cycles of the mean band-pass (for 80–250 Hz band-pass, the low-pass was 55 Hz). Subsequently, the power of the non-clipped signal was computed, and all events exceeding 4 SD from the mean were detected. Events were then expanded until the (non-clipped) power fell below 1 SD; short events (< 15 ms) were discarded. The pyramidal layer of the CA1 region was identified physiologically by increased unit activity and characteristic LFP patterns. The identification of dendritic sublayers was achieved by the application of current source density and independent component analysis to the LFPs^16,39,49,50^. To detect dentate spikes, we bandpass filtered (2 – 50 Hz) the LFP recorded from a hilus and a molecular layer location. We detected a positive deflection of LFP difference between the channels in the hilus and molecular layer. If the mean LFP value at the molecular layer during the dentate spike was lower than the baseline value (– 36 ms to –16 ms) by > 0.19 mV, the dentate spike passed the criteria and was included for later analysis. The peak time of the dentate spike was defined as the time when the dentate hilus wide-band LFP showed maximum value^30^.

### Backpropagation analysis

The regularly spaced and dense electrode arrays allow quantification of backpropagation properties of action potentials. Following clustering using Kilosort 2.5 and manual curation, backpropagation was assessed by first performing a search for above-threshold waveforms on electrodes near the cluster’s dominant channel, i.e. the channel with the largest spike amplitude approximating the soma location. The threshold was set to 12% of the minimum trough amplitude^31^. In some cases, troughs were detected at time points earlier than that of the channel with the largest spike amplitude. In such cases, the channel with the earliest peak was deemed to initiate the action potential. Finally, the selected channels’ locations and corresponding trough delays were fitted with linear regression to extract the slopes, which correspond to the inverse of the speed of backpropagation in time per unit distance (ms/mm). In cases where distinct slopes were detected above and below the putative soma location, two slopes were extracted accordingly. Cells were classified into those with backpropagation being above (ascending), below (descending), or both (curved).

### Mapping of the spike-LFP gamma oscillation coupling

Coupling of the spiking of single neurons to the LFP on each channel and at diverse gamma frequencies (30–200 Hz) was estimated using PLV statistics, i.e. the resultant length of the ensemble of phase values derived from the instantaneous phase of the band-pass filtered LFP signal in a 5 Hz band centered on the respective frequency of interest. For smoothing and visualization purposesa the adjacent four pixels within each location were averaged. This analysis was performed on raw data that were not processed by the real-time hardware, but offline. Raw electrode signals acquired with the SiNAPS Research Platform were post-processed to remove signal discontinuities associated with the regular calibration of the in-pixel DC frontend and to filter electrode signals in a frequency bandwidth ranging from 0.1 Hz to 5 kHz.

### Coherence maps in the gamma frequency

The LFP signals were filtered by a Gaussian band-pass filter (from 30 to 90 Hz), and coherence maps were constructed between manually selected LFPs at reference sites in different layers and the remaining 1023 channels (n = 100 one-second intervals during sharp wave ripples). Reference sites were: 2–2 channels in cortex and stratum pyramidale of CA3, one channel in stratum oriens, pyramidale and radiatum of CA1, granule cell and molecular layer of dentate gyrus). This procedure reliably outlined the anatomical layer boundaries across layers of the hippocampus and across regions (cortex and hippocampus)^16^. As for mapping spike-LFP gamma oscillation coupling, this analysis was performed on offline processed raw electrode signals that were acquired with the SiNAPS Research Platform.

### Statistical Analysis

Statistical analyses were performed with MATLAB functions or custom-made scripts. No specific analysis was used to estimate minimal population sample or group size, but the number of animals, sessions and recorded cells were larger or similar to those employed in previous related works^16,22,39^. The unit of analysis was typically identified as single neurons. In a few cases, the unit of analysis was sessions or animals, and this is stated in the text. Unless otherwise noted, non-parametric two-tailed Wilcoxon rank-sum (equivalent to Mann-Whitney U-test) or Wilcoxon signed-rank test was used. For multiple comparisons following ANOVA, Tukey’s honesty post-hoc test was employed. On box plots, the central mark indicates the median, bottom and top edges of the box indicate the 25th and 75th percentiles, respectively, and whiskers extend to the most extreme data points not considered outliers. Outliers are not displayed in some plots but were included in statistical analysis.

## Data Availability

The dataset will be available from the Buzsaki-lab data bank^51^, (https://buzsakilab.com/wp/database/) and G-Node repository (https://gin.g-node.org/antsiro/SiNAPS_8shank). MATLAB script packages used in the analysis of this study can be downloaded from https://github.com/MouseLand/Kilosort, https://phy.readthedocs.io/en/latest/ and https://github.com/buzsakilab/buzcode.
